# Nutritional status and growth of children and adolescents with and without cerebral palsy in eastern Uganda: A longitudinal comparative analysis

**DOI:** 10.1371/journal.pgph.0001241

**Published:** 2023-06-13

**Authors:** Lukia Hamid Namaganda, Carin Andrews, Fred Wabwire-Mangen, Stefan Peterson, Hans Forssberg, Angelina Kakooza-Mwesige

**Affiliations:** 1 CURIE Study Consortium, Iganga-Mayuge Health and Demographic Surveillance System, Iganga, Uganda; 2 Department of Epidemiology and Biostatistics, Makerere University, Kampala, Uganda; 3 Department of Women’s and Children’s Health, Uppsala University, Uppsala, Sweden; 4 Department of Women’s and Children’s Health, Karolinska Institutet, Stockholm, Sweden; 5 Department of Global Public Health, Karolinska Institutet, Stockholm, Sweden; 6 Department of Health Policy Planning and Management, Makerere School of Public Health, Kampala, Uganda; 7 Department of Pediatrics and Child Health, Makerere University, Kampala, Uganda; Penn State Health Milton S Hershey Medical Center, UNITED STATES

## Abstract

There is a need to understand the growth and burden of malnutrition in children with cerebral palsy (CP) in order to design appropriate inclusive nutrition strategies. We compared the nutritional status and four-year longitudinal growth of a population-based cohort of children and adolescents (C&A) with CP (n = 97; 2–17 years; 55/42 M/F), and an age and sex matched group without CP (n = 91; 2-17y; 50/41 M/F) in rural Uganda. The cohorts were assessed in 2015 and 2019 for weight, height, social demographic characteristics, and feeding related factors. Nutritional status was determined using the World Health Organization (WHO) Z-scores. Wilcoxon sign rank and Mann-Whitney tests were used to test within and between group differences. Multivariable linear regression was used to determine predictors of the change in growth. Approximately two thirds (62/97 (64%)) of C&A with CP were malnourished (with <-2SD in any of the WHO Z-scores), especially those with feeding difficulties (OR = 2.65; P = 0.032), and those who needed to be fed (OR = 3.8; P = 0.019). Both the CP and non-CP groups deviated negatively from the WHO reference growth curve for height, with a significantly slower growth in the CP group (median change score of height-for-age Z score (HAZ) between assessments = -0.80(-1.56, 0.31), p<0.01), than the non-CP group (median HAZ change score = -0.27(-0.92,0.34, p = 0.034). There was a statistically significant group difference in the median HAZ change score between the CP and non-CP groups (z = -2.21, p = 0.026). Severity of motor impairment measured by the Gross Motor Function Classification System (GMFCS-level) correlated negatively (r = -1.37,95%CI -2.67, -0.08) with the change in HAZ scores among the CP group. Children and adolescents with severe motor impairments exhibit an increased risk of malnutrition and growth retardation compared to their age matched peers without CP, which underscores the need to develop inclusive community-based nutrition strategies for children with cerebral palsy.

## Introduction

Children with cerebral palsy (CP), a common motor developmental disorder, are at increased risk of malnutrition and growth retardation [1, 2]. Children with CP living in Uganda face additional risks of undernutrition given the higher prevalence (33%) of stunting among under five year olds in eastern Africa compared to the global average (22%) [3]. How these compounding factors intersect is however not clear. The current estimate of the CP prevalence in low and middle income countries (LMICs) (3.1–3.7 per 1000 children) doubles that in high income countries (1.6–2.9 per 1000 children), [4] with 72–98% of children in LMICs reporting at least one form of under nutrition [5].

Studies in Africa on clinical cohorts of children with CP have shown a very high prevalence of malnutrition among children with CP, e.g., in Uganda 52% were malnourished and 38% stunted [6], and in North West Nigeria 83% were malnourished and 53% stunted [7]. Clinical cohort studies are informative but have the risk of selection bias and may not reflect the real situation, in particular in a low resource setting where many children with disabilities like CP have limited access to health services [8]. Therefore, it is important to gather population-based data. Recently established population-based CP registries in two other LMICS, Bangladesh and Nepal, showed that more than two thirds of children were malnourished [9, 10].

There is a multifaceted relationship between nutrition and disability since malnutrition may be both a cause and an effect of disability [11, 12]. The causal pathways of malnutrition in a child with CP may stem from several factors including: severity of the motor impairment and functional capability of the child [13]; inadequate dietary intake [14]; difficulties in feeding [15]; or community misconceptions and stigma which promote fewer meal times, less nutritious or smaller quantities of food for them [16]. The effect of malnutrition and disability in low resource settings are worsened by poverty, ignorance and limited access to health care services often leading to poorer health and in severe cases death. In a previous study we reported an excessively increased risk for premature death in a population based cohort of children and youth with CP in Uganda, especially among those with severe motor impairments and malnutrition [17]. Similar increased mortality has been reported in Malawi [18] and Bangladesh [19], other LMICs with a high prevalence of stunting.

In order to develop efficient and sustainable nutrition strategies for children with disability it is critical to first understand their nutritional challenges and growth patterns. Such strategies are urgently needed to reduce the premature mortality and to achieve many other sustainable development goals linked to nutrition [20]. In Uganda, the national child survival strategy [21], and national and international nutrition management guidelines, currently lack detailed specifications for children with disabilities [22]. This policy gap is partly due to limited evidence-based data preferably at a population level. Comparable estimates among typically developed children may offer a clear understanding of the burden of malnutrition in the CP population, which will inform targeted inclusive community-based nutrition guidelines with distinct requirements for children with disabilities. Therefore, in this study on a population-based cohort of children and adolescents with CP, we aimed to compare the nutritional status, and longitudinal growth of a cohort of children and adolescents with CP to those of an age and sex matched group without CP in a rural Ugandan setting. Specific objectives were to explore: 1) Group differences in nutritional status and feeding related factors at recruitment. 2) Group differences in growth over a 4-year period. 3) Potential influence of social demographic and feeding related factors on the nutritional status and longitudinal growth.

## Materials and methods

### Study design and participants

This was a population-based cohort study on children and adolescents (C&A) with and without cerebral palsy (CP) living in the Iganga-Mayuge Health and Demographic Surveillance Site (IM-HDSS) in eastern Uganda. The IMHDSS is an open population cohort resident within 13 peri-urban (near the Municipality) and 52 rural villages. The IMHDSS supports population level research including availability of a data-base of all households and trained research assistants who collect basic demographic data of residents in the area, through bi-annual census rounds since 2005. The IMHDSS population grows at an average annual rate of 4.8%, and in 2017, there were 94,568 individuals [23]. Data collection for this study was conducted in 2015 and 2019 at the IMHDSS as part of the research project “Cerebral palsy in Uganda: Risk factors, Intervention and Epidemiology (CURIE)”, which has resulted in a series of reports [17, 24, 25]. In 2015, the CURIE study used a three-stage screening procedure among 31,756 children and adolescents aged 2–17 years living in the IM-HDSS, and identified 97 C&A with CP [24]. Diagnosis of CP was set by the medical doctor, clinical officer and therapists using the Surveillance of CP in Europe (SCPE) guidelines [26]. The Ugandan and Swedish researchers further discussed and confirmed diagnosis via internet using video clips of each child [26]. A reference sample of 91 age and sex matched C&A without CP were also sampled from the same geographical area in 2015. Both groups of C&A were assessed twice; first in 2015 and a second time in 2019. We used a cross-sectional design to describe and compare the nutritional status of C&A with and without CP at the first assessment in 2015.

### Sample size estimation

Diggle et al.’s [27] formula was used to determine if the surviving participants at follow-up would enable us acquire an 80% powered study for comparison of two groups in a balanced design. We used an effect size of 0.5, and a 0.55 correlation of repeated measures in height for age z-scores [28] to estimate a minimum sample size of 54 participants per group required for the longitudinal analysis after adjusting for 10% attrition as follows:

$$\text{N}=\frac{2{\left(\text{z}\alpha +\text{z}\beta \right)}^{2}(1+\left(\text{n}-1\right)\rho )}{\text{n}{\left[\left(\mu 1-\mu 2\right)/\sigma \right]}^{2}}$$

$$\text{N}=\frac{2\left(1.96+.842\right)2\left(1+\left(2-1\right)\times .5\right)}{2\times \left(.5\right)2}=\left(15.7\right)\left(1.55\right)/\left[\left(2\right)\left(.25\right)\right]=48.67\;\text{per}\;\text{group}$$

Assumptions:

•zα = 1.96 2-tailed .05 hypothesis test •effect size (μ1−μ2)/σ = 0.5

•zβ = .842 power = 0.80 •n = 2 time points •ρ = 0.55 correlation of repeated measures in height for age z scores of children with CP [28]

After adjusting for attrition (10% at the second time point after the first), N = 48.67/0.9 = 54 per group.

### Study measurements and procedures of data collection

All C&A were clinically assessed by trained therapists, a clinical officer, medical doctors and a study nurse at the first assessment (2015), and at the second (2019) assessment, a different clinician re-assessed the weight, height, social demographic characteristics, and feeding related factors as had been done in 2015. The socio demographic characteristics included sex, age of the child, caregiver relationship to the child, level of education, marital status and monthly household income. Semi-structured questionnaires were used by the study nurse to collect data on feeding related factors such as feeding frequency, caregiver reported feeding problems, and feeding dependence. The gross motor function was classified by the therapist team using Gross Motor Function Classification System (GMFCS) [29], which classifies mobility on a 5-point ordinal scale, i.e., varying from level I (mild/independent) to level V (severe/ dependent on assistance for all mobility functions). All data collectors underwent two weeks of intense training including field tool pre-testing before study initiation. Supervision of the anthropometric measurements during home visits was done by one of the main researchers (NLH).

#### Anthropometric measurements

1. Weight was measured using a well calibrated SECA 813 digital scale (Seca Vogel & Halke GmbH &Co., Hamburg, Germany) with the child unclothed and readings recorded to the nearest 0.1 kg. When the child could not stand independently, the body weight was obtained by calculating the difference between the caregiver’s weight with and without the child.
2. Height was measured in centimetres (cm) using a portable stadiometer length measuring board with a headboard and sliding foot piece (Shorr Productions, LLC, Maryland, USA) and recorded to the nearest 0.1 cm for C&A who were able to stand flat-footed and erect. Those unable to stand had their length measured while lying down with a correction factor made to convert the measurement into height [30]. For C&A with significant musculoskeletal deformities (such as kyphosis, scoliosis or notable deformities in lower-limb flexion) or severe spasticity, height estimates were predicted from the knee height using published validated equations, most recommended in such circumstances due to depicting minimal errors relative to the measured height [31]. For each C&A the height/length was measured twice by a highly skilled nurse with an average obtained as the correct value. Care was taken to ensure that individuals with hair ornaments or braid buns did not have these items interfere with the height/length measurements.
3. Body mass index was calculated as weight (kg) divided by height in meters squared (m^2^).

### Data management and statistical analysis

We collected and entered the data electronically using the Open data tool kit (ODK) software. Stata version 14 (Statcorp, College Station, TX, USA) was used for all statistical analyses. Recorded weight and height were entered into WHO Anthro (2010) and Anthro-plus (2009) calculators, in which Height, Weight and Body Mass Index (BMI) Z-scores for age and sex were automatically calculated up to 19 years of age [32, 33]. The nutritional status of each child was assessed on the basis of Z-scores [34] as height-for-age Z score (HAZ), weight-for-age Z-score (WAZ) or BMI-for-age Z score (BMIZ). We defined malnutrition as severe when one or more of the anthropometric z-scores was <-3SD, and as moderate between -2SD and -3SD. The WAZ was only calculated for children up to 10 years. Stunted growth was defined as HAZ <-2SD. Associated impairments including visual, hearing, intellectual, and behaviour impairments, and the presence of seizures were recorded by i) a study nurse, ii) a medical officer, and iii) a therapist using pretested and pre-coded questionnaires to interview caregivers combined with standard clinical examinations. An impairment was confirmed if reported from at least two sources. We used a cross-sectional design to describe the sociodemographic and nutritional data from the first assessment in 2015, and to analyse the relationship by group (CP vs non-CP), and GMFCS levels (I-II vs III-V) in the CP group, using Pearson’s chi-square tests for independence. Descriptive analyses were used in addition to graphical representations.

To capture the longitudinal growth, we plotted the height and weight at the first assessment in 2015, and the second assessment in 2019 for every participant with respect to standard WHO growth charts [35]. The median and interquartile range (IQR) of the HAZ, WAZ and BMIZ scores were determined for the first and second assessment for each participant. The median and IQR was also calculated for the change score defined as the difference in scores between the first and second assessment. Wilcoxon sign rank tests were used to test the difference in median z-scores of repeated measurements within each of the CP and non-CP groups by age and gender, and by GMFCS levels (I-II vs III-V) in the CP group. We evaluated the difference in scores at a statistical significance level of p<0.05. A Bonferroni correction test was applied to correct for multiple tests within groups, and an adjusted alpha was generated. In order to test the difference in change scores between the CP and non-CP groups, a two sample Wilcoxon rank-sum (Mann-Whitney) test was used.

In order to explore the independent predictors of the change in HAZ, WAZ and BMIZ scores as outcome variables in the CP group, we used stepwise regression analysis to build linear regression models. First, bi-variable analyses were conducted to identify variables that correlated with the change in z-scores at p-value <0.25 [36]. Next, identified variables and those reported as relevant in literature were entered into a combined multiple linear regression model. Variables were considered statistically significant at p< 0.05 by the likelihood ratio test. We used complete case analysis to handle missing data on the change in z-scores as an outcome variable in the determination of independent predictor variables.

### Ethical considerations

This study was approved by the Higher Degrees Research and Ethics Committee of the School of Public Health, College of Health Sciences, Makerere University (REC 180074403), and the Uganda National Council for Science and Technology (protocol REF: HS1427ES). All the caregivers of the study participants under age 18 years provided informed written consent and assent was obtained whenever possible to participate in the 2015 and 2019 assessments.

## Results

### Study participants

**Fig 1:**
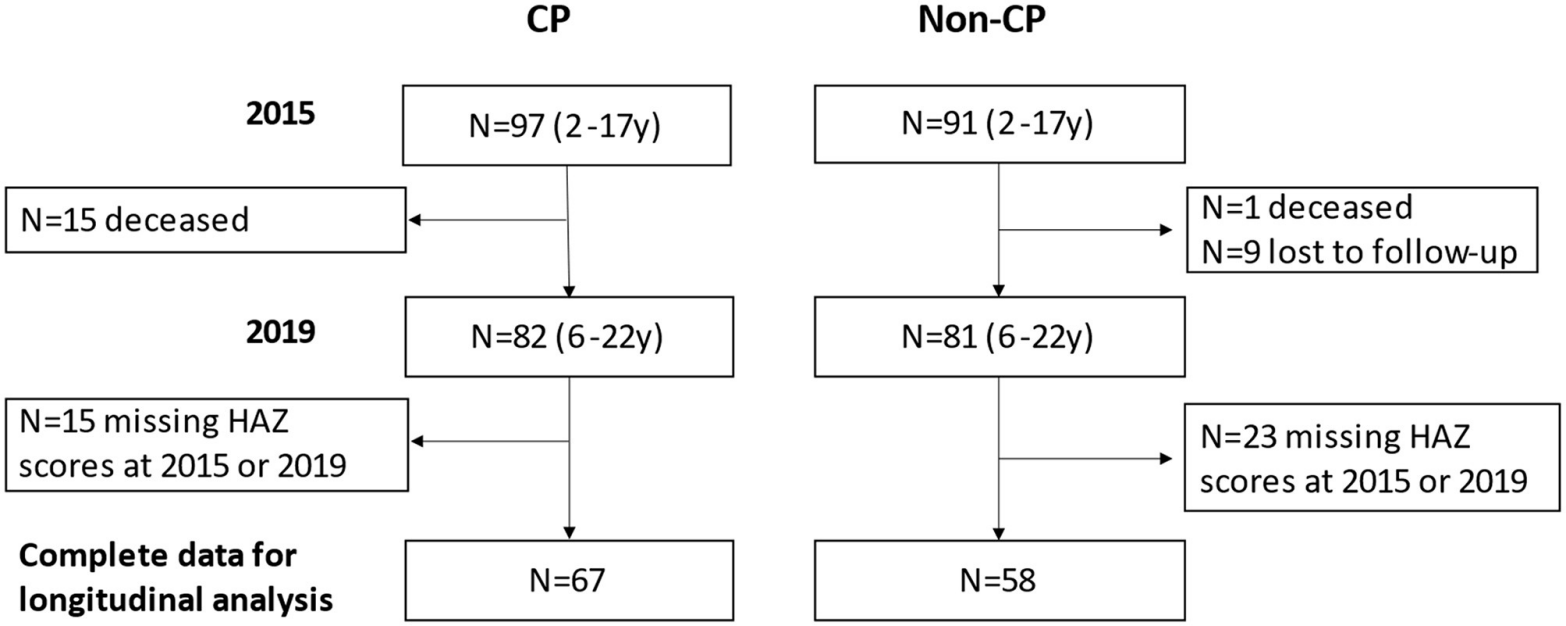
Study participants flow diagram.

Of the 97 CP and 91 Non-CP participants identified in 2015 at the first assessment, fifteen C&A with CP and one without CP were deceased after four years. Further nine C&A without CP were lost to follow-up. In addition, 15 C&A with CP and 23 C&A without CP had no calculated HAZ scores. Missing WAZ, HAZ, or BMIZ scores was due to: age limitation of WHO Growth Standard Charts (i.e., age above 10 years (no WAZ score) and age above 19 years (no HAZ and BMIZ scores), or missing recorded weight or height values at either the first or the second assessment. The longitudinal growth was analysed only among C&A with complete sets of HAZ data at both assessments (CP n = 67; without CP n = 58) as showed in Fig 1 below.

### Description of the CP and non-CP groups at recruitment

The socio-demographic characteristics of the two groups at the first assessment in 2015 are presented in Table 1. There were no statistically significant differences between groups except for marital status, with more caregivers never being married or separated in the CP group. In both groups, more than half of the households were living below the poverty level, earning less than 28 US dollars per month (i.e., <US$1 per day). A little over half of the participants were males, and almost two thirds of the primary caregivers were mothers in both groups, but with more grandmothers in the CP group. More than half of participants in the CP group were at GMFCS levels I-II.

**Table 1:** Social demographic characteristics of the CP and non-CP groups.

	CP group N = 97 (%)	Non-CP group N = 91 (%)	PearsonChi (χ^2^)	p-value
**Sex**			0.06	0.809
Male	55 (58)	50 (55)		
Female	42 (42)	41 (45)		
**Age**			0.36	0.835
2–5	34 (35)	30 (33)		
6–11	37 (38)	33 (36)		
12–17	26 (27)	28 (31)		
**GMFCS level**				
Level I-II	56 (60)			
Level III-IV	37 (40)			
Missing ^a^	4			
**Associated impairment** ^b^				
Yes	64(69)			
No	29(31)			
Missing ^a^	4			
**Marital status of caregiver**			**14.96**	**0.005**
Never married	15 (16)	2 (2)		
Married	61 (65)	76 (86)		
Separated	13 (14)	5 (6)		
Widowed	5 (5)	5 (6)		
Unknown	3	3		
**Caregiver relation to child**			24.09	0.000
Mother	70 (73)	57 (63)		
Father	04 (4)	12 (13)		
Other relative	02 (2)	13 (14)		
Grand mother	17 (18)	3 (3)		
Sibling	3 (3)	6 (7)		
Unknown	1	0		
**Monthly income** ^c^			5.01	0.286
< 100000shs	61 (69)	52 (71)		
100000 to 200000shs	20 (23)	14 (19)		
200000 to 500000shs	5 (6)	6 (8)		
> 500000shs	2 (2)	1 (1)		
Unknown	9	18		

Social and demographic characteristics of C&A with and without CP at the first assessment in 2015 matched by age and sex. Chi-square test was used to analyze differences between groups.

^a^Four children with CP were not classified according to the GMFCS and not assessed for associated impairments.

^b^ Associated impairments included visual and hearing impairments, intellectual disability, behavioral disorder and seizures.

^c^1 USD ≈3,500 Ugandan shillings (shs).

### Nutritional status and feeding related factors at the first assessment

Nutritional data of the two groups at the first assessment is presented in Table 2. There were large and highly significant differences between the CP and non-CP groups in all nutritional parameters (HAZ, WAZ, and BMIZ). Severe malnutrition was present in 45% (44) of the CP group compared with 8% (7) in the non-CP group, while 73% (65) had a normal nutritional status in the non-CP group compared with 36% (35) in the CP group. There were also significant differences within the CP group as children with more severe impairments (GMFCS level III-V) had poorer nutritional status than children with milder impairments (GMFCS levels I-II).

**Table 2:** Nutritional and feeding related characteristics of the CP and non-CP groups in 2015.

	CP N = 97(%)	Non-CP N = 91(%)	p-value	GMFCS I-II ^a^ N = 56 (%)	GMFCS III-V N = 37(%)	p-value
**Height for age (HAZ) Mean (SD)**	**-2.34(2.5)**	**-1.03(1.3)**	**0.000**			0.127
Normal >-2sd	39 (45)	67(75)		27(55)	11 (33)	
Moderate <-2sd to -3sd	18 (**21**)	15 (**17**)		12 (**24**)	5 (**15**)	
Severe <-3sd	29 (**34**)	7 (**8**)		10 (**20**)	17(**52**)	
Missing^b^	11	2		7	4	
**Weight for age (WAZ) Mean (SD)**	**-2.39(1.9)**	**-0.62(0.9)**	**0.000**			**0.000**
Obese>2sd	0 (0)	1 (2)		0 (0)	0 (0)	
Normal >-2sd to 2sd	32 (51)	50 (91)		24 (77)	7 (24)	
Moderate <-2sd to -3sd	7 (**11**)	4 (**7**)		3 (**10**)	4 (**14**)	
Severe<-3sd	24 (**38**)	0 (0)		4 (**13**)	18 (**62**)	
Missing	0	0		0	0	
Above 10 years ^b^^,^ ^c^	34	36		25	8	
**BMI for age (BMIZ) Mean(SD)**	**-1.09(2.3)**	**-0.09(1.2)**	**0.000**			**0.001**
Obese >2sd	4 (5)	2 (2)		3 (7)	1 (3)	
Normal >-2sd to 2sd	50 (60)	82 (93)		34 (74)	15 (45)	
Moderate <-2sd to -3sd	7 (**8**)	4 (**5**)		4 (**9**)	1 (3)	
Severe <-3sd	22 (**27**)	0 (**0**)		5 (**11**)	16 (**48**)	
Missing^b^	14	3		10	4	
**Nutritional status** ^d^			**0.000**			**0.000**
Normal >-2sd	35 (36)	65 (73)		26 (46)	9 (24)	
Moderate <-2sd to -3sd	18 (**19**)	17 (**19**)		14 (**25**)	4 (**11**)	
Severe <-3sd	44 (**45**)	7 (**8**)		16 (**29**)	24 (**65**)	
Missing	0	2		0	0	
**How often fed in 24 hrs**			0.528			0.425
Once	0 (0)	0 (0)		0 (0)	0 (0)	
Twice	11 (11)	15 (16)		9 (16)	2 (5)	
Thrice	75 (77)	62 (68)		42 (75)	31 (84)	
Four times	8 (8)	9 (10)		4 (7)	2 (5)	
More than 4 times	3 (3)	5 (5)		1 (2)	2 (5)	
**Self-feed**			**0.000**			**0.000**
Yes, skilfully	56 (58)	91 (100)		45 (80)	10 (27)	
Yes, but unskilled	20 (21)	0 (0)		10 (18)	9 (24)	
No, must be fed	21 (22)	0 (0)		1 (2)	18 (49)	
**Feeding difficulties**			**0.000**			**0.000**
Yes	33 (34)	0 (0)		8 (15)	22 (59)	
No	63 (66)	91 (100)		47 (85)	15 (41)	
Missing	1	0		1	0	

Nutritional and feeding related characteristics of C&A with and without CP at the first assessment in 2015.

^**a**^ Four children with CP were not classified according to GMFCS.

^b^ Missing due to unrecorded height or weight.

^**c**^ WAZ scores only for those above 10 years.

^d^ Nutritional status: Severe <-3sd in any of HAZ, WAZ, BMIZ; Moderate -2sd to -3sd in any of HAZ, WAZ, BMIZ. Pearson chi-square test was used to test the observed difference between groups with level of significance at p<0.

There were significant differences in feeding characteristics between groups. All C&A without CP (100%) fed skilfully (with feeding tools and by self) compared to 58% of C&A with CP. One third of C&A with CP had feeding difficulties, while no C&A without CP had any difficulties in feeding. There was no group difference in how often the children were fed; the majority were fed at least three times a day. Within the CP group, skilful self-feeding was less frequent and feeding difficulties more common at GMFCS levels III-V than at GMFCS levels I-II (Table 2). The likelihood of being malnourished was significantly higher among those with feeding difficulties (OR = 2.65; P = 0.032), and those who needed to be fed (OR = 3.8; P = 0.019) (Table 3).

**Table 3:** Factors associated with malnutrition among C&A with and without CP at the first assessment.

	CP N = 97 n(%)	Adj. odds ratios (95%CI)	p-value	Non-CP N = 91 n(%)	Adj. odds ratios (95%CI)	p-value
**How often fed in 24h**						
Twice	11 (11)	ref		14 (15)	ref	
Thrice	75 (77)	1.2(0.32,4.24)	0.811	72 (79)	0.56(0.16,1.98)	0.371
Four times	8 (8)	1.3(0.26,7.18)	0.719	05 (06)	1	
> 4 times	3 (3)	1.3(0.20,8.12)	0.797	0		
**Self-feed**						
Yes, skillfully	56 (58)	ref		91		
Yes, but unskilled	20 (21)	1.6(0.55,4.64)	0.392	0		
No, must be fed	21 (22)	3.8(1.25,11.5)	0.019*	0		
**Feeding difficulties**						
No	63 (66)	ref		91		
Yes	33 (34)	2.65(1.08,6.45)	0.032*	0		
Unknown	1			0		
**Monthly income**						
<100,000shs	61 (64)	ref		52	ref	
>100,000shs	27 (36)	0.90(0.36,2.26)	0.829	21	1.43(0.45,4.53)	0.545
Unknown	9			18		

Factors associated with malnutrition among C&A with CP at the first assessment. Odds ratios after bivariate and multivariate logistic regression modelling of malnutrition as an outcome of feeding related factors and household income at the first assessment in 2015. Malnutrition was defined as <-2SD in any of HAZ, WAZ, or BMIZ.

*p<0,05 statistical significance

### Longitudinal change in the nutritional status in C&A with and without CP

#### Growth of height (HAZ)

**Fig 2:**
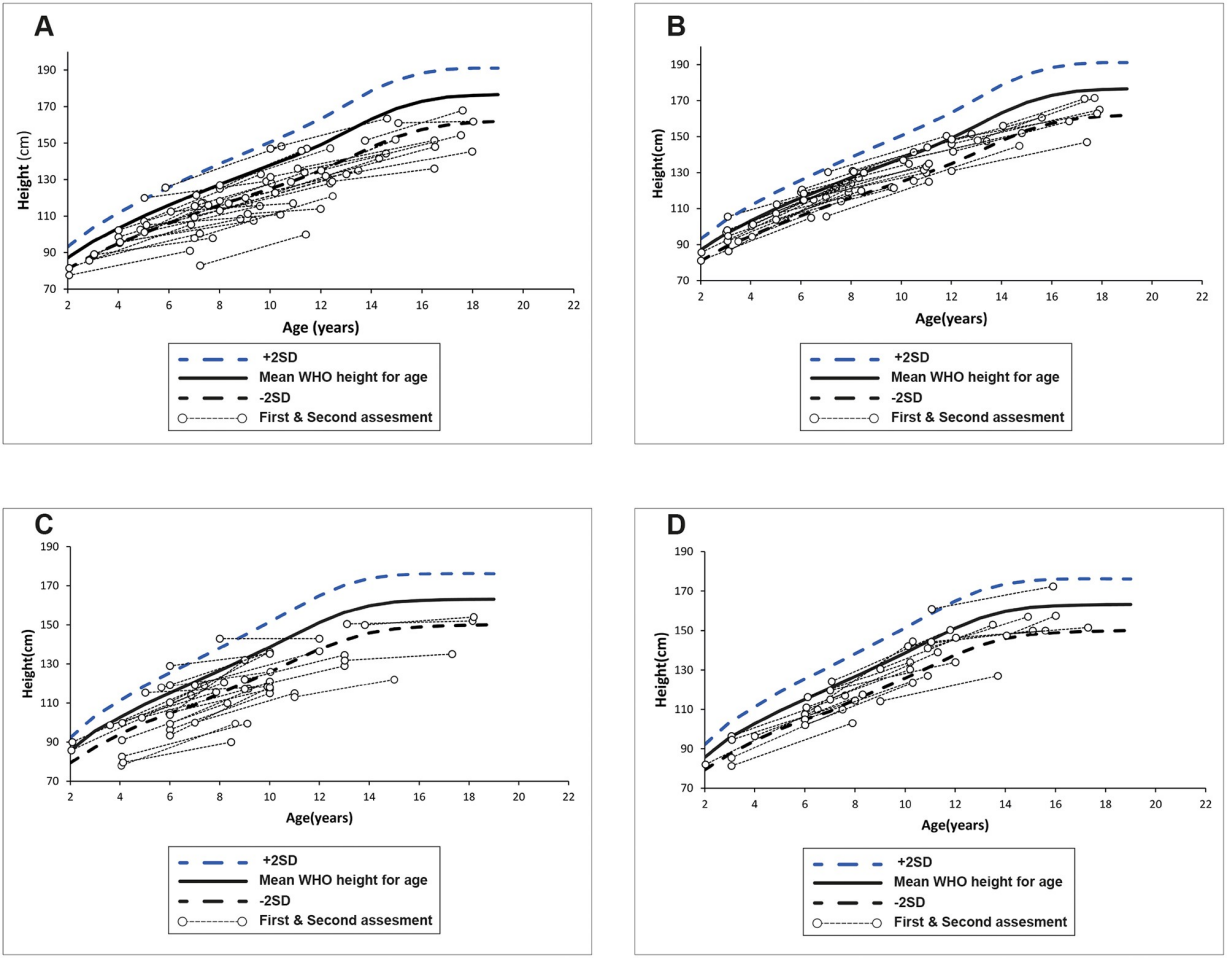
Height for age trajectory for C&A with and without CP plotted against WHO growth reference. A, Males 2–19 years with CP; B, Males 2–19 years without CP; C, Females 2–19 years with CP; D, Females 2–19 years without CP. https://doi.org/10.1093/pch/15.2.84.

The height of every C&A was plotted against WHO growth reference curves at the first and second assessment (Fig 2A–2D). The curves illustrate that many males and females with CP were below the mean of the reference height at the first assessment and that their growth trajectories were less steep than the WHO growth curves.

The change in HAZ scores between the first and second assessment for both groups is presented in Table 4. Significant negative HAZ change scores were exhibited within both the CP and non-CP groups, indicating that both groups had a lower growth rate than the WHO growth standards. Furthermore, a Mann-Whitney U test showed a statistically significant difference in the median HAZ change scores between the CP and Non-CP groups (z = -2.21, p = 0.026), with a higher negative HAZ change score among the CP (mean = -0.93, median = -0.8) than non-CP (mean = -0.24, median = -0.27) groups, thus demonstrating a slower growth in the CP group. Among C&A with CP, a negative HAZ change score was highest in the youngest (2–5 years median change score = -1.01, p = 0.009), and oldest age groups (median change score = -1.40; p = 0.006), and among C&A with more severe GMFCS levels III–V (change score = -1.25; p = 0.0007). There were similar significant negative change scores in both females and males with CP.

**Table 4:** Change in Height for Age z-scores (HAZ) in the CP and non-CP group.

Height for age CP-group	N	2015 Median (IQR)	2019 Median (IQR)	Change score Median (IQR)	z	p
All children	67	-2.24(-3.47, -0.86)	-2.99(-4.19, -1.61)	-0.80(-1.56, 0.31)	-3.59	**0.000** *
**Sex**						
Male	40	-2.48(-3.10, -1.21)	-2.97(-4.08, -1.82)	-0.85(-1.70, -0.33)	-2.77	**0.005** *
Female	27	-1.63(-4.15, -0.55)	-3.10(-4.36, -1.27)	-0.73(-1.40,0.30)	-2.30	**0.021** *
**Age(years) 2015**						
2–5	26	-2.38(-2.99, -0.90)	-2.97(-5.17, -1.61)	-1.01(-1.83, -0.50)	-2.58	**0.009** *
6–11	31	-2.47(-4.15, -0.87)	-2.93(-3.95, -1.53)	-0.58(-1.15, -0.70)	-1.37	0.170
12–15	10	-1.20(-2.77, -0.55)	-3.47(-4.21, -1.98)	-1.40(-2.15, -0.73)	-2.70	**0.006** *
**GMFCS level 2015**						
GMFCS I-II	44	-1.92(-2.97, -0.86)	-2.24(-3.92, -1.20)	-0.58(-1.31, -0.40)	-1.99	0.046*
GMFCS III-V	23	-2.62(-4.52, -0.44)	-3.82(-5.70, -2.69)	-1.22(-3.36, -0.31)	-3.39	**0.001** *
**Height for age Non-CP group**						
All children	58	-1.11(-2.12, -0.42)	-1.47(-2.11, -0.69)	-0.27(-0.92, 0.34)	-2.11	**0.034** *
**Sex**						
Male	33	-1.14(-2.14, -0.28)	-1.41(-2.04, -0.58)	-0.28(-0.73, -0.35)	-1.62	0.105
Female	25	-1.09(-1.85, -0.43)	-1.70(-2.13, -0.88)	-0.27(-1.01, 0.26)	-1.39	0.161
**Age(years) 2015**						
2–5	24	-1.81(-2.25, -0.99)	-1.53(-2.15, -0.92)	0.02(-0.35,0.73)	0.70	0.483
6–11	26	-0.74(-1.30, 0.10)	-1.39(-1.80, -0.65)	-0.56(-1.01,0.10)	-2.85	**0.004** *
12–15	8	-0.82(-1.59, -0.20)	-1.71(-2.32, -1.04)	-0.37(-1.73, -0.02)	-1.68	0.092
^a^ **Between group difference**	125	-1.13	-1.52	-0.52	-2.21	0.026

Change in height for Age z-scores (HAZ) in the CP and non-CP group. The median Height for age z-scores (HAZ) at the first (2015) and second (2019) assessment, and the Change score (the difference of the two assessments) are presented for the CP and Non-CP group. Wilcoxon sign rank tests were used to test the difference between the first and second assessment.

^a^Mann-Whitney test was used to estimate the between group differences in the CP versus non-CP groups.

Adjusted p-value = 0.005 after correcting for multiple comparisons(k = 9). Original statistical significance at

*p<0.05.

Multiple linear regression modelling was used to analyse factors that correlated with longitudinal growth in height of C&A with and without CP (Table 5). Among the CP group, the severity of the motor impairment (GMFCS level) correlated with the change in HAZ score (r = -1.53; p = 0.023), while there were no correlations between feeding related factors and longitudinal growth, although feeding difficulty was associated with malnourishment at the first assessment. The monthly income of less than 200,000 shillings (≈ < 57USD) was significantly correlated with a change in HAZ scores in the non-CP group (p = 0.027), and almost significant in the CP group (p = 0.054).

**Table 5:** Factors associated with the change in HAZ among the CP and non-CP groups.

	CP group	Adj. coefficients (95%CI)	Adj. P-values	non-CP group	Adj. coefficients (95%CI)	Adj. P-values
	Freq. n (%)			Freq. n (%)		
**Age** (years)						
2–5	26 (36)	ref		24 (41)	ref	
6–11	31 (49)	0.54(-1.85,0.77)	0.413	26 (45)	-0.18(-0.85,0.49)	0.594
12–15	10 (15)	-1.21(-2.92,0.49)	0.158	08 (14)	0.17(-0.84,1.18)	0.736
**Sex**						
Male	40 (63)	ref		33(57)	ref	
Female	27 (37)	-0.39(-1.58, 0.78)	0.501	25(43)	-0.08(-0.70,0.53)	0.788
**GMFCS level**						
Level I-II	44 (66)	ref				
Level III-V	23(34)	-1.37(-2.67, -0.08)	**0.038** *			
**Associated impairment**						
No	24 (36)	ref				
Yes	42(64)	-0.15(-1.44,1.12)	0.810			
Unknown	01					
**Feeding difficulties**						
No	46 (70)	ref		58 (100)		
Yes	20 (30)	-0.06(-1.45,1.57)	0.937	00		
Unknown	1					
**Self-feeding**						
Yes, skillfully	42 (63)	ref		46 (79)	ref	
Yes, but unskilled	13 (19)	0.07(-1,57,1.42)	0.918	04 (07)	-0.72(-2.09,0.64)	0.292
No, must be fed	12 (18)	-1.13(-3.13,0.86)	0.258	08 (13)	0.19(-0.87,1.25)	0.721
**Malnourished** ^a^						
No	40 (60)	ref		42 (72)	ref	
Yes	27 (40)	0.92(0.33,2.18)	0.147	16 (28)	1.15(0.44,1.86)	0.002*
**Monthly income**						
>100000	37(60)	ref		36 (71)	ref	
100,000 to 200000	18(30)	1.38(-0.02,2.80)	0.054	10(20)	1.01(0.12,1.91)	**0.027** *
200,000 to 500,000	5(08)	0.80(-1.52,3.14)	0.492	04 (8)	-0.74(-2.10,0.63)	0.284
>500,000	1(02)	-1.96(-6.84,2.90)	0.422	01(2)	-0.39(-2.95,2.18)	0.763
Unknown	6			7		

Factors associated with the change in HAZ among the CP and Non-CP groups. Multiple linear regression modeling of the change in HAZ scores of 67 C&A with CP and 58 C&A without CP, respectively, and factors assessed at the first examination in 2015. Adjusted coefficients were derived from multivariate analysis.

^a^ Malnourished defined as <-2SD in any of HAZ, WAZ or BMIZ in 2015.

*Statistical significance at p<0.05.

#### Growth of weight and body mass index

The weight for age of every C&A was plotted against WHO growth standard charts at the first and second assessment (S1A & S1B Fig). The curves illustrate that many males and females with CP were below the weight reference at the first assessment and had a less steep trajectory than the reference weight curve (which ends at 10 years). Particularly females with CP seemed to have a flatter growth curve at older ages than peers without CP. A statistically significant negative WAZ change score was observed among males with CP (change score = -0.75, p = 0.023), while females exhibited a small positive insignificant change (change score = 0.35, p = 0.609) (S1 Table). The Non-CP group had a significant negative change score (change score -0.23, p = 0.49, driven by the male group (-0.50, p = 0.57). Multiple linear regressions did not reveal any association of feeding related factors or family income with the WAZ change score among the CP group.

Change of the BMIZ score for both groups is presented in S2 Table. The CP group had a significant negative change score (-2.13, p = 0.333) driven by males and those in the youngest age group. Insignificant negative changes in BMIZ scores were observed among those most severely impaired (GMFCS III-V). In the Non-CP group only the youngest age group had a negative BMIZ change score (-2.71, p = 0.006). Neither feeding related factors nor family income were associated with the BMIZ change score in the CP group after multivariate linear regression modelling.

## Discussion

The main findings of this study were that two thirds (64%) of C&A with CP were malnourished in comparison to WHO growth standards, and that malnutrition was significantly more prevalent in this group than in an age and sex matched non-CP reference group living in the same rural area in Uganda. The longitudinal follow up confirmed that the CP group had a significantly slower growth compared with the non-CP group, while both groups deviated negatively from the WHO reference growth curve for height. In the CP group, the severity of the motor impairment (GFMCS level III-V), feeding difficulties, and inability to self-feed was associated with poor nutritional status at the first assessment, while only GMFCS-level correlated to growth in height during the 4-year follow-up period.

More than half (55%) of the C&A with CP in this rural population were stunted (<-2SD HAZ), a marker of chronic malnutrition and growth retardation. In a previous hospital cohort from the Ugandan National referral hospital in Kampala, we found that 38% of the children with CP were stunted [6]. At that time, we suspected this could be a misrepresentation of the population in view of selection bias inherent in hospital cohorts with potentially more severe forms of CP and referrals from other parts of the country. In contrast, our present study suggests that malnutrition is even more common (64%) among the rural population of C&A with CP in Uganda portraying higher rates of stunting (55%). Such high rates are in agreement with more recent population based studies from two other low income countries, Bangladesh and Nepal, reporting stunting in 73% and 66%, respectively [9, 10]. The high prevalence of stunting in the Ugandan CP child population compared with a much lower prevalence in HICs [37–39] is most likely an effect of the low resource setting with prevalent malnutrition among the general child population. The UNICEF/WHO/World Bank group reports the globally high prevalence of stunting in Eastern Africa at 35% [3], while 25% of our reference group was stunted, which is comparable with the national prevalence in Uganda of 29% [40]. During the last decades there has been a dramatic global reduction of stunting in the general population, for example from 49% to 35% in Eastern Africa, and from 50% to 33% in South Asia [3], and in Uganda from 48% (1988) to 29% (2016) [41]. Interventions related to this decline have included poverty reduction, improved access to maternal care, and malaria infection-reduction [42]. Our study findings suggest that these strategies have not benefitted C&A with CP who need a more focused approach. This may largely be explained by the scarcity of disability specific recommendations in the available international guidelines to the management of child malnutrition, based on which most national guidelines are modelled [22]. The urgency of more effective actions is exemplified by 25 times higher excessive mortality rate among C&A with CP than the general child population in rural Uganda [17], in which severe impairments and severe malnutrition were strong risk factors for premature mortality.

### Factors associated with the four-year growth

Our study results are in agreement with evidence showing that the severity of the motor impairments is a strong predictor of malnutrition in children with CP [39]. The overall dominating cause of retarded growth is insufficient intake of calories and proteins, which in turn can be caused by poor oral-motor functions such as chewing and swallowing [43, 44]. We found that C&A with more severe motor impairments (GMFCS III-V) had more feeding difficulties and more often needed assistance when feeding resulting in lower food intake as a result of slow rates of feeding, extended feeding times, and excessive spillage of food. This may be due to caregiver’s lack of knowledge on how to feed their child with CP effectively or of how to teach the child to self-feed bearing in mind that some of these children may need special seating or positioning. Challenging meal times could also have led to increased stress levels for the caregiver and the child, resulting in inadequate food intake [15]. Considering the serious and documented fatal consequences of malnutrition in this group [17, 19], there is a need to develop interventions that maximize the child’s food intake. This could include altering postural positioning, increasing the frequency of feeding, adjusting food texture and optimizing food intake by high calorie supplements, proteins and micronutrients [44, 45]. In HIC, the introduction of tube feeding and gastrostomy has proven beneficial to both child and family [46], but the technique is not feasible in most low resource settings due to high risks of infections [47] and negative caregiver perceptions [48]. Children with CP spend most of their time with caregivers, hence culturally acceptable and affordable nutrition interventions that involve caregivers as primary actors are vital in low resource settings.

In our study, growth retardation (negative change of HAZ) was observed in the youngest (2-5y) and oldest (12-18y) age groups with CP. Based on evidence from HICs, it is known that growth in height among individuals with CP worsens with age independent of nutritional status [49, 50] which has initiated a discussion around the development of special reference growth curves for children with CP. The results of this study, and the previous finding that malnourishment was most common in children above 5 years of age in the hospital cohort in Kampala [6] suggests a similar age effect on growth in C&A with CP in Uganda. A novel finding not earlier described is the pronounced growth retardation in the youngest age group in our cohort. In addition, there were some significant negative change scores for WAZ and BMIZ in the youngest (2-5y) children in both the CP and Non-CP groups. While stunting (HAZ) is the effect of chronic malnutrition, underweight (WAZ) and thinness (BMIZ) reflects more recent undernutrition. Our finding confirms that undernutrition is common among both the CP and Non-CP groups during the first five years of life, and that it affects the early growth of all children, especially those with CP. One reason stunting and underweight are less prevalent in the middle age group in our study, could be the high mortality of the most impaired and malnourished children above five years of age [17]. The most malnourished children in our study are thus part of the youngest age group, but not in the older age groups since they may be deceased. There is limited and contradicting information about growth in relation to age in C&A with CP from other LMICs; in Ghana stunting was associated with older ages [5]; in Bangladesh with the middle age group 5–10 years [9]; and in Western China there was no difference among age groups [38]. Obviously, more population based studies from LMIC are needed to understand the effect of age on growth.

Our study suggests that sex was not associated with the HAZ change score, and both males and females with CP had significant height growth retardation. In contrast, males with CP, but not females, had significant negative change score for both WAZ and BMIZ. There is little information on the effect of sex on malnutrition in children with CP in the literature. In a large CP cohort from California, the difference in weight in comparison to the general population, was larger for high functioning males with CP than for females [50]. No sex differences were reported from the population based studies in Bangladesh and Nepal [9, 10]. So far research data are inconclusive and due to cultural differences girls and boys may be treated differently in different settings. These findings warrant further research to explore the gender specific determinants of growth among children with CP in the rural Ugandan setting.

A household monthly earning of more than 200,000 shillings was positively correlated with growth of the C&A in the non-CP group and almost significant in the CP-group. This corresponds to studies from other LMICs like Botswana, Bangladesh, Nepal and Vietnam showing more prevalent malnourishment in families with low income and other socioeconomic risk factors [9, 10, 51, 52]. The relatively small effect in the CP group might be the low income-levels in the majority of the families i.e., 69% had an income less than 1 USD per day, 23% earned between 1 USD and 2 USD per day, and only 8% of households had an income above 2 USD per day. The differences in income might have been too small to reveal any impact in the CP group while it is obvious that most families in our study were living under tough economic conditions known to enhance malnutrition.

### Study strengths and limitations

This study encompasses several strengths. It is the first longitudinal population-based study investigating the nutritional status of C&A with CP in sub-Saharan Africa. In addition to using international reference growth curves, we compared with an age and sex matched reference group living in the same area, making it possible to disentangle the environmental impact of the low resource setting and the nutritional challenges inflicted by the disability. By following the two cohorts during a four-year period we could for the first time assess the longitudinal growth of a CP cohort in an LMIC. The use of internationally standardized diagnoses and assessment tools further made it possible to compare with studies from other countries.

Among the limitations is the relatively small number of participants and missing data that further diminished the sample size and increased the possibility of bias, in particular for the longitudinal analyses. However, our sample was still sufficient to yield a powered comparative study given the sample size requirements indicated. Furthermore, we collected data at only two time points which precluded detection of nonlinearities in the data overtime.

Another limitation was the technique (knee height or length) used to measure height, which was challenging to conduct in some children with CP owing to fixed joint contractures, spasticity, involuntary muscle spasms, poor cooperation owing to associated cognitive impairments or inability to stand. Since height is a factor in calculation of BMI, an inaccurate height may have skewed the BMI results.

This study was based on mothers/caregivers reported practices rather than a detailed dietary intake report, and so prone to recall and response bias. It was also beyond the scope of the study to conduct a detailed environmental assessment or assess the quality of local health and nutrition services offered in the area. Additionally, this study was observational and thus causality could not be confirmed. This necessitates the need for controlled experimental longitudinal studies to confirm the reported associations with nutritional outcome over time. Furthermore, although CP diagnosis with the three-stage community survey method included the SCPE criteria adapted to the local context, it should be noted that CP profiles may vary by socioeconomic status, and thus validation in the application of SCPE guidelines in the low resourced Ugandan context is warranted.

## Conclusion

Our study showed that malnutrition and poor growth is higher among C&A with CP compared to their age and sex matched peers without CP, and that feeding difficulties and severe motor impairments are related factors. Existing community management of malnutrition programs should include detailed and specific guidelines for children with disabilities like CP, as has been done for their typically developed peers.

### Recommendations

It is crucial that the existing nutrition policies and guidelines are appropriately revised to equally benefit children with disabilities like CP. Given that no one anthropometric measure of nutritional status is a ‘gold standard’, future studies should explore how well the respective indicators detect children with CP at high risk of mortality/morbidity, notwithstanding the urgent need for CP specific growth charts. This will inform, clarify and validate their usability across different settings. Furthermore, the mid upper arm circumference (MUAC) measure was not included in our assessment of nutrition status. The utility of the MUAC in screening and case detection for acute malnutrition in children especially for those at highest risk of adverse health outcomes has been reported to be better than other indicators [53]. It may therefore be advantageous for programmes to further evaluate its reliability as a practical tool in routine screening for children with CP.

Specifications on feeding and positioning, growth monitoring, dietary and energy requirements by ambulatory status should be included as this has been proven effective [54]. Implementation requires a multidisciplinary approach that includes, training on physical rehabilitation, and knowledge of disability among community health workers, and integration of trained caregivers of children with CP into the lay community health workforce or village health teams. The involvement of caregivers in applicable and user-friendly training and support programs on feeding practices of children with CP could also be advocated to enhance their children’s nutritional status [55]. These efforts may reduce stigma, and improve the identification and referral of disabled children within communities for timely management of feeding and/or motor impairments with malnutrition as has been successfully done among the typically developed children. Further exploratory studies to determine whether the caloric requirements in children with CP match those of their non-CP counterparts in LMICs should also be undertaken.

## Supporting information

S1 Fig A. Weight for age trajectory for males 2–21 years with CP plotted against WHO growth reference. https://doi.org/10.1093/pch/15.2.84, B. Weight for age trajectory for males 2–21 years without CP plotted against WHO growth reference. https://doi.org/10.1093/pch/15.2.84, C. Weight for age trajectory for females 2–21 years with CP plotted against WHO growth reference. https://doi.org/10.1093/pch/15.2.84, D Weight for age trajectory for females 2–21 years without CP plotted against WHO growth reference. https://doi.org/10.1093/pch/15.2.84.
(TIF)

S1 Table Change in weight for age z-scores among CP and Non-CP groups. The median weight for age z-scores (WAZ) at the first (2015) and second (2019) assessment, and the Change score (the difference of the two assessments) are presented for the CP and Non-CP group. Wilcoxon sign rank tests were used to estimate differences between the first and second assessment. Statistical significance at *p<0.05.
(TIF)

S2 Table Change in body mass index for age z-scores among CP and Non-CP groups. The median Body mass index for age z-scores (WAZ) at the first (2015) and second (2019) assessment, and the Change score (the difference of the two assessments) are presented for the CP and Non-CP group. Wilcoxon sign rank tests were used to estimate differences between the first and second assessment. statistical significance at *p<0.05. **Two children were missing weight measurements in 2019, one refused to stand on scale while the other was too sick to be moved for weight measurements.
(TIF)
