# Supplementary figures and images for: Nutritional status and growth of children and adolescents with and without cerebral palsy in eastern Uganda: A longitudinal comparative analysis
Source: PLOS Glob Public Health. 2023 Jun 13;3(6):e0001241. doi: 10.1371/journal.pgph.0001241 (PMC10263332; doi:10.1371/journal.pgph.0001241)

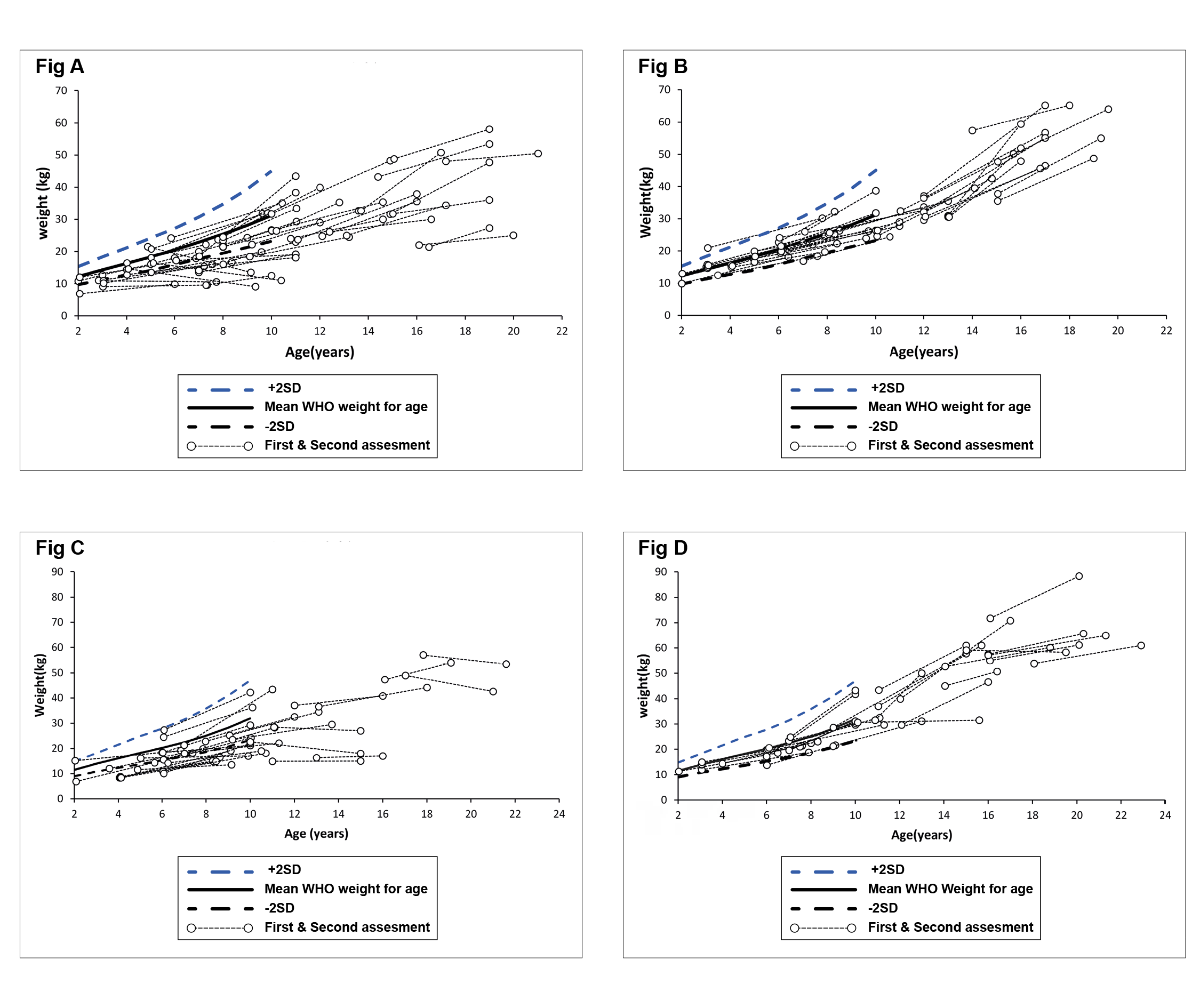

Supplement: S1 Fig — A. Weight for age trajectory for males 2–21 years with CP plotted against WHO growth reference. https://doi.org/10.1093/pch/15.2.84, B. Weight for age trajectory for males 2–21 years without CP plotted against WHO growth reference. https://doi.org/10.1093/pch/15.2.84, C. Weight for age trajectory for females 2–21 years with CP plotted against WHO growth reference. https://doi.org/10.1093/pch/15.2.84, D Weight for age trajectory for females 2–21 years without CP plotted against WHO growth reference. https://doi.org/10.1093/pch/15.2.84. (TIF) [file pgph.0001241.s001.tif]

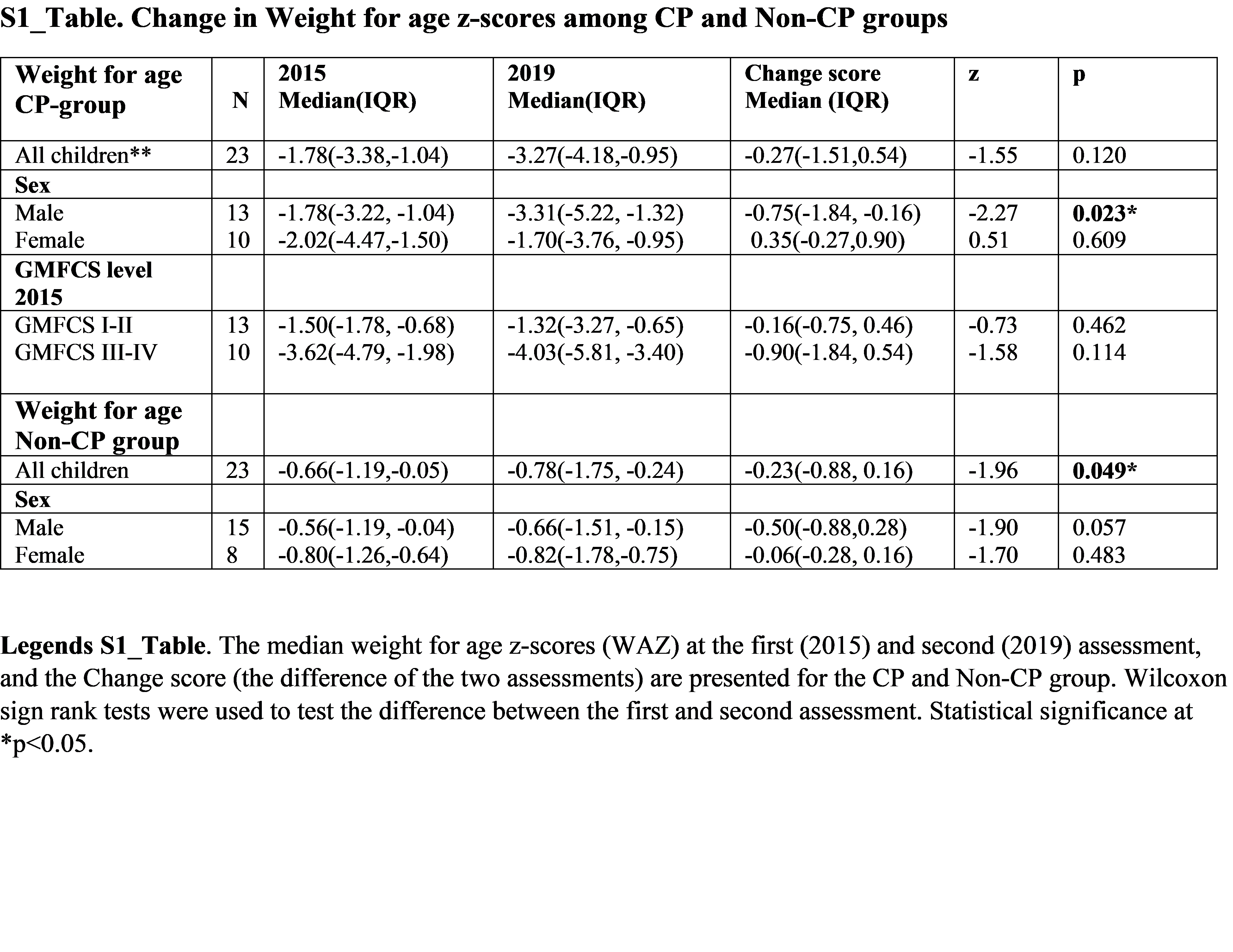

Supplement: S1 Table — The median weight for age z-scores (WAZ) at the first (2015) and second (2019) assessment, and the Change score (the difference of the two assessments) are presented for the CP and Non-CP group. Wilcoxon sign rank tests were used to estimate differences between the first and second assessment. Statistical significance at *p<0.05. (TIF) [file pgph.0001241.s002.tif]

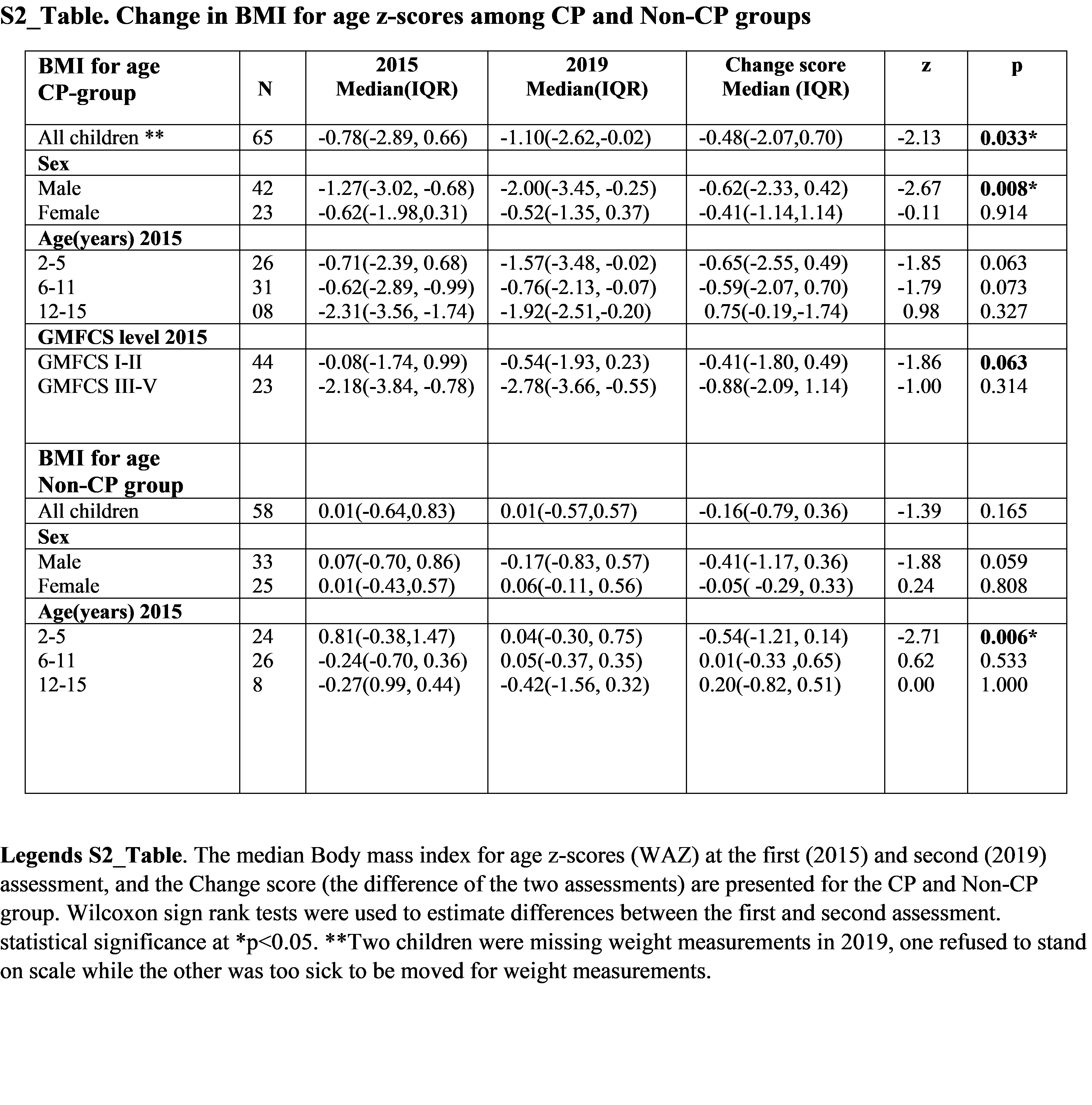

Supplement: S2 Table — The median Body mass index for age z-scores (WAZ) at the first (2015) and second (2019) assessment, and the Change score (the difference of the two assessments) are presented for the CP and Non-CP group. Wilcoxon sign rank tests were used to estimate differences between the first and second assessment. statistical significance at *p<0.05. **Two children were missing weight measurements in 2019, one refused to stand on scale while the other was too sick to be moved for weight measurements. (TIF) [file pgph.0001241.s003.tif]
